# Simulated Optimum Sowing Date for Forage Pearl Millet Cultivars in Multilocation Trials in Brazilian Semi-Arid Region

**DOI:** 10.3389/fpls.2017.02074

**Published:** 2017-12-08

**Authors:** Rafael D. Santos, Kenneth J. Boote, Lynn E. Sollenberger, Andre L. A. Neves, Luiz G. R. Pereira, Carolina B. Scherer, Lucio C. Gonçalves

**Affiliations:** ^1^Embrapa Semi-Arid, Petrolina, Brazil; ^2^Department of Agronomy, University of Florida, Gainesville, FL, United States; ^3^Embrapa Dairy Cattle, Juiz de Fora, Brazil; ^4^Animal Sciences Department, Federal University of Minas Gerais, Belo Horizonte, Brazil

**Keywords:** climate change, crop models, cultivars competition, dry lands, DSSAT, rainfed agriculture

## Abstract

Forage production is primarily limited by weather conditions under dryland production systems in Brazilian semi-arid regions, therefore sowing at the appropriate time is critical. The objectives of this study were to evaluate the CSM-CERES-Pearl Millet model from the DSSAT software suite for its ability to simulate growth, development, and forage accumulation of pearl millet [*Pennisetum glaucum* (L.) R.] at three Brazilian semi-arid locations, and to use the model to study the impact of different sowing dates on pearl millet performance for forage. Four pearl millet cultivars were grown during the 2011 rainy season in field experiments conducted at three Brazilian semi-arid locations, under rainfed conditions. The genetic coefficients of the four pearl millet cultivars were calibrated for the model, and the model performance was evaluated with experimental data. The model was run for 14 sowing dates using long-term historical weather data from three locations, to determine the optimum sowing window. Results showed that performance of the model was satisfactory as indicated by accurate simulation of crop phenology and forage accumulation against measured data. The optimum sowing window varied among locations depending on rainfall patterns, although showing the same trend for cultivars within the site. The best sowing windows were from 15 April to 15 May for the Bom Conselho location; 12 April to 02 May for Nossa Senhora da Gloria; and 17 April to 25 May for Sao Bento do Una. The model can be used as a tool to evaluate the effect of sowing date on forage pearl millet performance in Brazilian semi-arid conditions.

## Introduction

In Brazil, the semi-arid region comprises 95 million hectares of which only 3% is suitable for irrigation, leaving an immense dryland area to be exploited if sustainable production practices can be identified and implemented (Martins et al., 2003). Under these conditions, forage production represents one possible alternative.

Within this context, pearl millet is a candidate feed source for agricultural adaptation in dry regions as it is a tropical plant possessing the C4 photosynthetic pathway and it has tolerance to drought, heat and low soil pH (Maiti and Wesche-Ebeling, 1997). Because of its adaptability to harsh conditions, millet can be grown in areas that are unfavorable to other crops such as maize (Singh and Singh, 1995). Although, several studies have evaluated the potential of pearl millet as forage for ruminants in dry regions (Messman et al., 1992; Hill et al., 1999), data on its management are limited, especially about ideal sowing dates. For semi-arid regions, the planting date decision is important not only because of its effect on yield but also the need to minimize the risk of establishment failures and to decrease cost and labor required for replanting.

Crop simulation models can be useful tools for the evaluation of alternative management options for a particular location, including planting dates (Tsuji et al., 1998; Ruiz-Nogueira et al., 2001; Saseendran et al., 2005). Furthermore, models that have been developed and validated with local experimental data can be valuable tools for extrapolating these experimental results to other years and other locations (Matthews et al., 2002). Crop simulation models integrate the interdisciplinary knowledge gained through experimentation and technological innovations in the fields of biological, physical, and chemical science relating to agricultural production systems (Soler et al., 2007; Andarzian et al., 2015). These models have been used to investigate the performance of different pearl millet cultivars at a range of sowing dates in relation to different soil-climate scenarios (Pale et al., 2003; Shivsharan et al., 2003a; Soler et al., 2008). The Decision Support System for Agrotechnology Transfer (DSSAT) is a comprehensive decision support system for assessing management options (Tsuji et al., 1998; Jones et al., 2003; Hoogenboom et al., 2015). This system includes the Cropping System Simulation Model (CSM)-CERES-Pearl Millet (Jones et al., 2003; Hoogenboom et al., 2015), which is a process-oriented, dynamic crop simulation model that simulates crop growth, development, and yield.

The overall goal of this study was to evaluate the performance of the CSM-CERES-Pearl Millet model for simulating growth, development, and forage accumulation of four pearl millet cultivars and to determine optimum sowing dates for pearl millet forage yield under rainfed conditions in three Brazilian semi-arid locations.

## Materials and Methods

### Experimental Sites

The experiments were carried out in 2011, under rainfed conditions, at three locations in the Brazilian semi-arid region: Bom Conselho, Pernambuco State; Nossa Senhora da Gloria, Sergipe State; and Sao Bento do Una, Pernambuco State. The experiment in Bom Conselho (09°10′ S, 36°40′ W, elevation of 654) was conducted at a private property, located in a region in which the soil type is Xanthic ferralsol sand clay (Santos et al., 2013) with a fine texture and pH of 4.5 to top soil. The climate is typically semi-arid with an annual rainfall of 431 mm and average maximum and minimum temperatures of 31 and 19°C, respectively. The experiment in Nossa Senhora da Gloria (10°13′ S, 37°25′ W, elevation of 291 m) was conducted at the Semi-Arid Experimental Station of the Brazilian Agricultural Research Corporation (EMBRAPA). The soil type in this region is a Eutrophic podzol clay loam (Santos et al., 2013), with an average depth of 1.5 m and pH of 6.1 to top soil. The climate is typically semi-arid with an annual rainfall of 710 mm and average maximum and minimum temperatures of 32 and 20°C, respectively. The experiment in Sao Bento do Una (08°31′ S, 36°26′ W, elevation of 614 m) was conducted at an experimental station of the Agronomic Institute of Pernambuco (IPA), where the soil was a sandy loam Eutrophic leptosol with medium texture and pH of 6.6 to top soil. The megathermal climate at this site is typically semi-arid with an annual rainfall of 655 mm and average maximum and minimum temperatures of 32.6 and 11.6°C, respectively.

### Pearl Millet Cultivars Evaluated

Four pearl millet cultivars, IPA Bulk1BF, BRS 1501, CMS-03 and CMS-01, were used in the experiment. IPA Bulk1BF was developed by Agronomic Institute of Pernambuco (IPA). It produces to 6.0 Mg ha^-1^ forage accumulation under rainfed conditions. It takes 50–60 days to reach flowering during winter. It is recommended mainly for forage production in Brazilian semi-arid regions (Rodrigues and Pereira Filho, 2010). The Brazilian Agricultural Research Corporation (EMBRAPA) developed and released the cultivars BRS 1501, CMS-03, and CMS-01 that are adapted to areas with risks of drought conditions. They have forage accumulation averaging of 8.0 Mg ha^-1^ and good grain production potential (2.5 Mg ha^-1^). For these cultivars, flowering occurs 50 days after planting (Pereira Filho et al., 2003). The four cultivars were chosen because of their similar duration to maturity and because they are grown under rainfed conditions. The cultivars are already being grown by farmers, and, therefore, could easily be promoted for use under water deficit conditions once their performance is established.

### Treatments, Experimental Design, and Crop Management

The experiments were conducted under rainfed conditions during the rainy season from May to August 2011. Treatments were the four cultivars replicated five times in a randomized complete block design. Plots measured 10.5 m^2^ (5 m × 2.1 m), with seed sown to a depth of 3 cm in four rows (on 0.70 m centers). The sowing dates were 17 May 2011, 1 June 2011, and 4 June 2011 for Sao Bento do Una, Nossa Senhora da Gloria, and Bom Conselho, respectively. At sowing the following fertilization was applied: 30 kg ha^-1^ nitrogen (as ammonium sulfate), 450 kg ha^-1^ triple superphosphate and 100 kg ha^-1^ potassium chloride. Two side-dress fertilizations were applied, the first on the 30th day, and the second on the 60th day after plant emergence, with the dose equivalent to 30 kg ha^-1^ of nitrogen (as ammonium sulfate).

Cultivars were harvested when at least 60% of plants in each plot reached the Zadoks scale of 85, which means that plants were at the dough stage of grain maturity (Zadoks et al., 1974). Harvests were manually at 5 cm stubble height, and only the two central rows in each plot were harvested, with the remainder being discarded. The harvested crop from each plot was collected and weighed to estimate fresh forage accumulation ha^-1^. After chopping a representative sample from each plot, a 400 g sub-sample was oven-dried at 55°C for 48 h to determine dry matter concentration and forage accumulation of the four cultivars.

### Climate, Soil, and Crop Data

The minimum input data set required for DSSAT version 4.6 to simulate crop growth was discussed in detail by Jones et al. (2003). Input data required for the models are crop management information, cultivar-specific parameters (genetic coefficients), soil properties and daily weather variables of the study areas.

Weather data from 2011, for three locations, were obtained from automated weather stations, including daily average, maximum and minimum temperatures, precipitation, daily rainfall, evaporation, and relative humidity (**Table 1**). For trials that were performed on agricultural research stations, on-station data were available. For the experiment that was performed on-farm (Bom Conselho), weather data were obtained from the recording station nearest to the farm (at a distance of 4 km). For analysis of the different simulated sowing dates 15 years of weather records were used for each site, including rainfall data that were obtained from automated weather stations. The other historical weather data including air temperatures, wind speed, evaporation and relative humidity were obtained from NASA-POWER database (Stackhouse et al., 2015). The method of Priestley and Taylor (1972) was used to estimate the evapotranspiration during the simulations.

**Table 1:** Meteorological data during the experimental period for the selected locations.

Location^¶^	Rainfall^†^	Rain^§^	Temperature^‡^			Evaporation^§^	RH^††^
			Maximum	Minimum	Mean		
Bom Conselho	44	223	32	22	26	180	75
Nossa Senhora da Gloria	61	282	27	18	22	217	77
Sao Bento do Una	66	304	27	18	21	243	85

^†^*Rainfall occurrence in days*.

^§^*Rain and evaporation in mm*.

^‡^*Temperature in °C*.

^††^*RH, average relative humidity (%)*.

^¶^*Experimental period: Bom Conselho – from 4 June to 25 August 2011; Nossa Senhora da Gloria – from 1 June to 30 August 2011; Sao Bento do Una – from 17 May to 09 August 2011*.

The crop data collected in 2011 include phenology dates (sowing and anthesis dates), forage accumulation and its components (separated into leaves, stems, and panicles). The forage accumulation was measured at the dough stage since these cultivars were grown for forage production. Physiological maturity was estimated based in previous publications (Duraes et al., 2003; Pereira Filho et al., 2003).

Soil P, K, Al, Ca, Mg, organic matter, pH, and initial soil moisture concentration data to a depth of 0.3 m were collected in 2011 before sowing. For simulations for each field, only soil series and surface soil texture were available from Santos et al. (2013). Pedon data from the Harmonized World Soil Database (HWSD) (Fischer et al., 2008) were used to provide estimates of bulk density, organic carbon, and percent sand, silt, and clay in each layer. The methods of Saxton et al. (1986) were used to estimate volumetric soil water content at lower and upper limits and saturated hydraulic conductivity for each soil layer. We assumed a maximum soil profile depth of 1.0 m for Bom Conselho and Sao Bento do Una, and set layer depths to 5, 15, 30, 60 and 100 cm, while for Nossa Senhora da Gloria was used soil profile depth of 1.5 m and set layer depths to 5, 15, 30, 60, 100, and 150 cm. Soil albedo (SALB), runoff potential (SLRO), and drainage rate (SLDR) were estimated according to the procedures outlined in DSSAT documentation (Hoogenboom et al., 2015). The soil fertility factor (SLPF) was assumed to be 1.0 in all simulations. The soil surface evaporation limit (SLU1) was set to 6.0 mm d^-1^ for all sites. Initial soil water content was assumed to be at field capacity in all simulations and full recharge at the time of sowing. For N fertilization management simulations, ammonium sulfate was used as fertilizer, where N was applied at planting (30 kg ha^-1^), and as a sidedressing application before flowering (20 kg ha^-1^).

### Weather Time Series Analysis

The three locations have characteristics typical of semi-arid tropical regions, i.e., a hot and dry climate that is highly limited in its hydrologic resources, particularly due to low precipitation and high evaporation rates. These sites have two well-defined seasons, namely, the rainy season, lasting between 3 and 4 months during the summer and fall, and the dry season, lasting for the remainder of the year, and this was the main determinant to sowing window.

**FIGURE 1:**
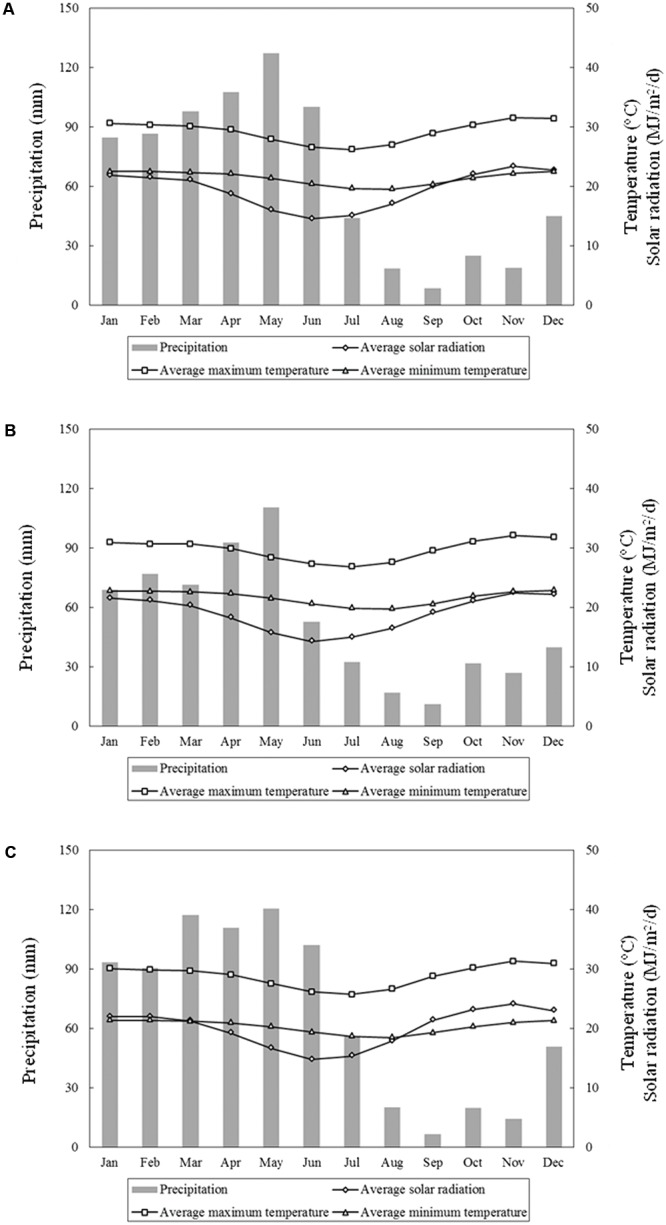
Average monthly precipitation, average maximum and minimum temperature, and average solar radiation for Bom Conselho **(A)**, Nossa Senhora da Gloria **(B)**, and Sao Bento do Una **(C)** for 1997–2011.

The analysis of the series of 15 years (1997–2011) of weather records for Bom Conselho, showed that the average monthly maximum temperatures were always greater than 26°C, and the average monthly minimum temperatures were always higher than 19.5°C (**Figure 1A**). The rainfall during the 2011 crop growing season (223 mm) was above the 15-year average (163 mm) and there were more rainy days (44) than the long-term average (24 days).

For Nossa Senhora da Gloria, the 15-year average of weather records showed that the highest maximum average temperatures and maximum solar radiation occurred in November (32 and 23°C, respectively) while the highest minimum average temperature occurred in December (22.5°C) (**Figure 1B**). With respect to precipitation, the highest monthly values were observed between January and June, with a maximum for May (111 mm). The 2011 growing season in Nossa Senhora da Gloria was characterized by a higher amount of rainfall (257 mm) and rainy days (61) than the 15-year long-term average growing season (102 mm and 27 days).

Similarly to Nossa Senhora da Gloria, the Sao Bento do Una weather records showed that the highest maximum average temperatures and maximum solar radiation occurred in November (31 and 24°C, respectively) while the highest minimum average temperature occurred in December (21°C) (**Figure 1C**). The rainy season, on average, started in January and ended in July, with a maximum precipitation of 121 mm in May. The 2011 growing season for Sao Bento do Una showed a similar amount of rainfall as the average for 15 years, with a total rainfall of 304 mm from 66 rainy days compared with the 15-year average total precipitation of 298 mm from 43 rainy days.

### Model Calibration and Evaluation

The CSM-CERES-Pearl Millet model of the DSSAT Version 4.6.1 (Jones et al., 2003; Hoogenboom et al., 2015) was used to evaluate the performance of pearl millet under the different sowing dates. The CSM-CERES-Pearl Millet model in V4.6.1 was modified and improved for temperature, water deficit, and tillering response based on extensive data from India and Africa (K. J. Boote, personal communication, 2015). Site-specific calibration of cultivar traits and evaluation of model performance are pre-conditions for using models for other locations than where they were developed (Jones et al., 2001; Van Ittersum et al., 2003). The primary objective of model calibration was to adapt the model parameters to local environmental conditions (e.g., soil types and weather conditions) and crop cultivars so as to gain a good overall agreement between simulated and observed values. The CSM-CERES-Pearl Millet model includes nine cultivar-specific coefficients that require modification for new cultivars not previously used with the crop model. Six specific cultivar coefficients were adjusted for pearl millet during the evaluation process: P1 – Thermal time from seedling emergence to the end of the juvenile phase (expressed in degree days above a base temperature of 10°C) during which the plant is not responsive to changes in photoperiod; P5 – Thermal time (degree days above a base temperature of 10°C) from beginning of grain filling (3–4 days after flowering) to physiological maturity; G1 – Scaler for relative leaf size on main stem; GT – Tillering coefficient; G4 – Scaler for partitioning of assimilates to the panicle (head) and PHINT – Phylochron interval; the interval in thermal time (degree days) between successive leaf tip appearances. These genetic coefficients were calibrated manually and determined in sequence, starting with the phenological development coefficients and followed by the crop growth coefficients, because of the dependence of the latter coefficients on the performance of the vegetative and reproductive development simulations (Hoogenboom et al., 1992).

Different statistical indices were employed, including Coefficient of Determination (*r*^2^), absolute and normalized root mean square error (RMSE) (Loague and Green, 1991), and Index of Agreement (*d*-Stat) (Willmott et al., 1985). Normalized RMSE gives a measure (%) of the relative difference of simulated versus observed data. The simulation is considered excellent when the normalized RMSE is less than 10%, good if the normalized RMSE is greater than 10% and less than 20%, fair if normalized RMSE is greater than 20 and less than 30%, and poor if the normalized RMSE is greater than 30% (Jamieson et al., 1991). According to the *d*-Stat, the closer the index value is to one, the better the agreement between the two variables that are being compared (Willmott et al., 1985). The combination of coefficients that resulted in the highest *d*-Stat and the smallest RMSE between observed and simulated values were selected as the final cultivar coefficients.

Forage accumulation data were analyzed by a mixed model approach with cultivars and locations as a fixed effect, random effects of blocks, and residual random error using the MIXED procedure of SAS Version 9.1 statistical program (SAS 2002).

### Optimum Sowing Dates

An analysis of the effect of different sowing dates on forage accumulation of pearl millet was conducted using 15 years of weather records for each site. In this study, 14 different sowing dates were simulated using the seasonal analysis tool of DSSAT Version 4.6.1, under rainfed conditions.

The sowing dates started on 1 January and were repeated every 15 days until 15 July. These dates were selected because this period is the regional rainy season, which coincides with the growth window for forage crops such maize and sorghum. However, due to the limitation of available water, there is a significant variation for regional planting window. Assumptions for determining the sowing window were that the opening sowing window was the first date on which 85% of the maximum forage accumulation could be obtained, and the closing sowing window was the last date for which 85% of the maximum forage accumulation could be obtained.

## Results

### Evaluation of the CSM-CERES-Pearl Millet Model

The values for cultivar coefficient P1 showed some variation, ranging from 75.0 to 118.9 degree days. The genetic coefficient P5 also had a significant and large variation, varying from 102.6 degree days for cultivar BRS 1503 to 377.0 for cultivar CMS-03. The value of G1 for IPA Bulk1BF and CMS-03 was 1.0, while the G1 values for BRS 1501 and CMS-01 were 0.5 and 1.2, respectively. The value of GT was 1.0 for all four cultivars. The cultivar reproductive partitioning coefficient G4 was 0.83 for the cultivar IPA Bulk1BF, 1.02 for BRS 1501, 0.65 for CMS-03 and 0.77 for CMS-01. The phyllochron interval coefficient (PHINT) value for all four cultivars was 43.0 degree days (**Table 2**).

**Table 2:** Genetic coefficients of pearl millet cultivars calibrated in DSSAT.

Cultivar coefficient description	IPA Bulk1BF	BRS 1503	CMS-03	CMS-01
P1 – Thermal time from seedling emergence to the end of the juvenile phase (expressed in degree days above a base temperature of 10°C) during which the plant is not responsive to changes in photoperiod	118.9	102.9	75.0	117.4
P2O – Critical photoperiod or the longest day length (in hours) at which development occurs at a maximum rate	12.31	13.00	12.20	12.45
P2R – Extent to which phasic development leading to panicle initiation (express in degree days) is delayed for each hour increase in photoperiod above P2O	174.1	157.4	160.1	149.2
P5 – Thermal time (degree days above a base temperature of 10°C) from beginning of grain filling (3–4 days after flowering) to physiological maturity	180.0	102.6	377.0	261.4
G1 – Scaler for relative leaf size on main stem	1.00	0.50	1.00	1.20
G4 – Scaler for partitioning of assimilates to the panicle (head).	0.83	1.02	0.65	0.77
PHINT – Phylochron interval; the interval in thermal time (degree days) between successive leaf tip appearances	43.0	43.0	43.0	43.0
GT – Tillering coefficient, equivalent to G1, but on tillers	1.00	1.00	1.00	1.00
G5 – Potential grain size, mg	11.0	11.0	11.0	11.0

After calibration, the model was able to predict the number of days from planting to anthesis and forage accumulation for the four pearl millet cultivars grown in all three locations during the 2011 growing season (**Tables 3**, **4**). At the three locations phenology varied among cultivars, since for all four cultivars, the period from planting to anthesis ranged from 50 to 56 days.

**Table 3:** Forage accumulation for pearl millet under rainfed conditions at three locations in Brazil, as measured and simulated after calibration.

Cultivars	Forage accumulation				
	Measured (kg ha^-1^)			Simulated (kg ha^-1^)	
	**Bom Conselho**				
IPA Bulk1BF	9650			10000	
BRS 1501	7650			7620	
CMS-03	11500			10400	
CMS-01	10580			10160	
	
	**Nossa Senhora da Gloria**				
	
IPA Bulk1BF	12050			11920	
BRS 1501	9750			9540	
CMS-03	14090			13700	
CMS-01	13180			12800	
	
	**Sao Bento do Una**				
	
IPA Bulk1BF	12610			13360	
BRS 1501	9870			10830	
CMS-03	14110			14100	
CMS-01	14010			13920	
	
	**RRMSE**^†^	***d*-Stat**^§^	***r*^2‡^**	**SEM^††^**	***P*^§§^**
	
IPA Bulk1BF	4.2	0.96	0.93	346.3	<0.001
BRS 1501	4.4	0.98	0.96		
CMS-03	5.0	0.94	0.99		
CMS-01	2.6	0.98	0.99		

^†^*RRMSE, normalized root mean square error (%)*.

^§^*d-Stat, Wilmot’s Index of Agreement*.

^‡^*r^*2*^, coefficient of determination*.

^††^*SEM, standard error of mean*.

^§§^*P, probability (if treatments differ at *P* < 0.05)*.

**Table 4:** Anthesis date for pearl millet under rainfed conditions at three locations in Brazil, as measured and simulated after calibration.

Cultivars	Anthesis				
	Measured (DAP)			Simulated (DAP)	
	**Bom Conselho**				
	
IPA Bulk1BF	53			53	
BRS 1501	50			50	
CMS-03	50			50	
CMS-01	54			55	
	
	**Nossa Senhora da Gloria**				
	
IPA Bulk1BF	55			54	
BRS 1501	54			52	
CMS-03	54			51	
CMS-01	56			52	
	
	**Sao Bento do Una**				
	
IPA Bulk1BF	54			58	
BRS 1501	53			56	
CMS-03	53			56	
CMS-01	55			57	
	
	**RRMSE^†^**	***d*-Stat**^§^	***r*^2‡^**	**SEM^††^**	***P*^§§^**
	
IPA Bulk1BF	4.4	0.19	0.37	0.67	0.01
BRS 1501	4.9	0.67	0.50		
CMS-03	4.6	0.64	0.17		
CMS-01	4.8	0.63	0.10		

^†^*RRMSE, normalized root mean square error (%)*.

^§^*d-Stat, Wilmot’s Index of Agreement*.

^‡^*r^*2*^, coefficient of determination*.

^††^*SEM, standard error of mean*.

^§§^*P, probability (if treatments differ at *P* < 0.05)*.

For Bom Conselho, the simulation exactly reproduced the observed days from planting to anthesis for the cultivars IPA Bulk1BF, BRS 1501, and CMS-03. For the cultivar CMS-01 observed, and simulated intervals were similar, 54 and 55 days, respectively. The average observed forage accumulation for the four cultivars was 9850 kg ha^-1^ and the corresponding average simulated forage yield was 9540 kg ha^-1^.

For Nossa Senhora da Gloria the observed number of days from planting to anthesis ranged from 54 to 56 days, while the simulated number of days to anthesis ranged from 51 to 56 days. At this site, the average observed and simulated forage accumulation values were 12270 and 11990 kg ha^-1^, respectively.

For Sao Bento do Una the observed number of days from planting to anthesis ranged from 53 to 55 days, while the simulated number of days to anthesis ranged from 56 to 58 days. The average observed forage accumulation for the four cultivars for this location was 12650 kg ha^-1^ while average simulated yield was 13050 kg ha^-1^.

The values of normalized RMSE and *d* for anthesis ranged from 4.4 to 4.9% and from 0.19 to 0.67, respectively. It is important to note that a given set of genetic coefficients for a cultivar were optimized across all three sites. For all three locations, the normalized RMSE for yield ranged from 2.6 to 5% and the value for *d* ranged from 0.94 to 0.98, the model was well-capable of simulating yields across the three sites.

There was not location effect or location × cultivar interaction. For forage accumulation, the *P*-value was 0.85, while for anthesis *P*-value was 0.88.

### Sowing Date Analysis

The sowing date analysis using 15 years of weather data (1997–2011) for Nossa Senhora da Gloria showed that the best sowing date for millet depends, in part, on the cultivar that will be used. However for Bom Conselho and Sao Bento do Una, all pearl millet cultivars had similar trends for the best sowing date.

**FIGURE 2:**
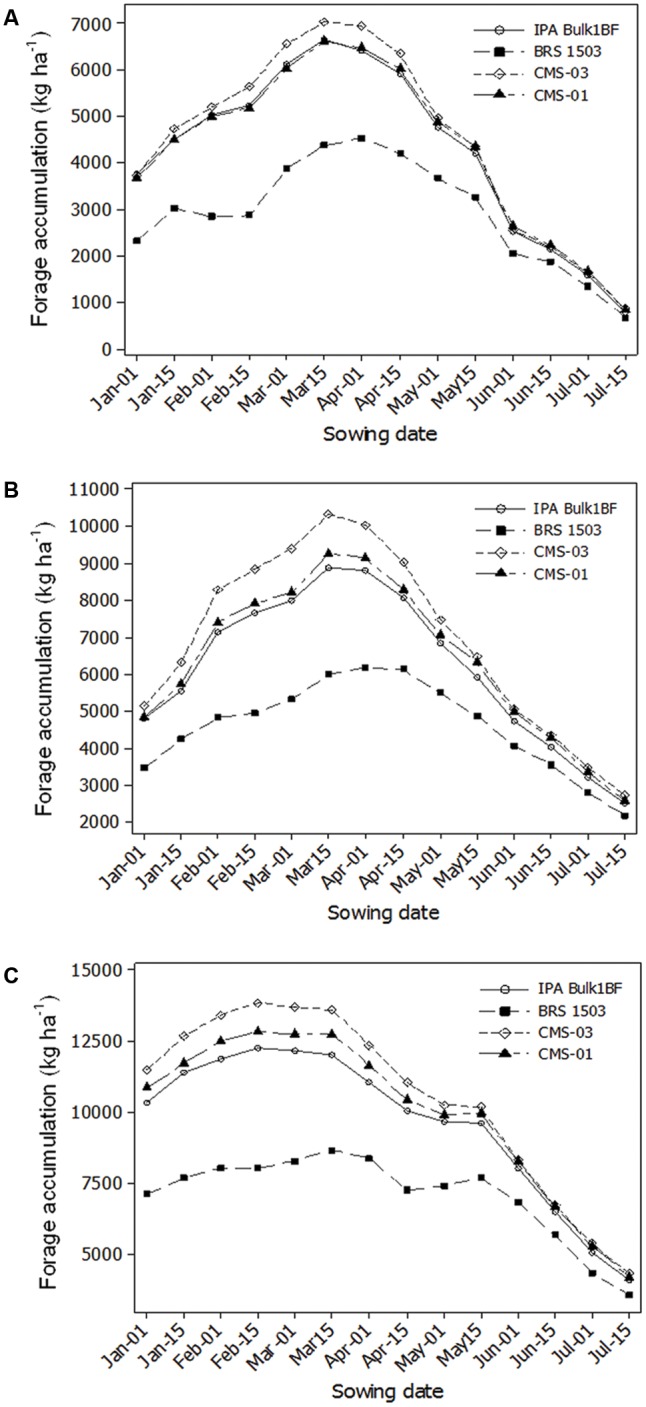
Simulated pearl millet forage accumulation for different cultivars at different sowing dates for Bom Conselho **(A)**, Nossa Senhora da Gloria **(B)**, and Sao Bento do Una **(C)**.

On the rising slope of yield versus sowing date, the average slope was 37, 54 and 29 kg ha^-1^ d^-1^ during the period prior to the peak yield. The slope of decline after peak yield with delayed sowing was 45, 51 and 58 kg ha^-1^ d^-1^, for Bom Conselho, Nossa Senhora da Gloria and Sao Bento do Una, respectively (**Figure 2**).

Individual points represent the four cultivars and planting dates.

**FIGURE 3:**
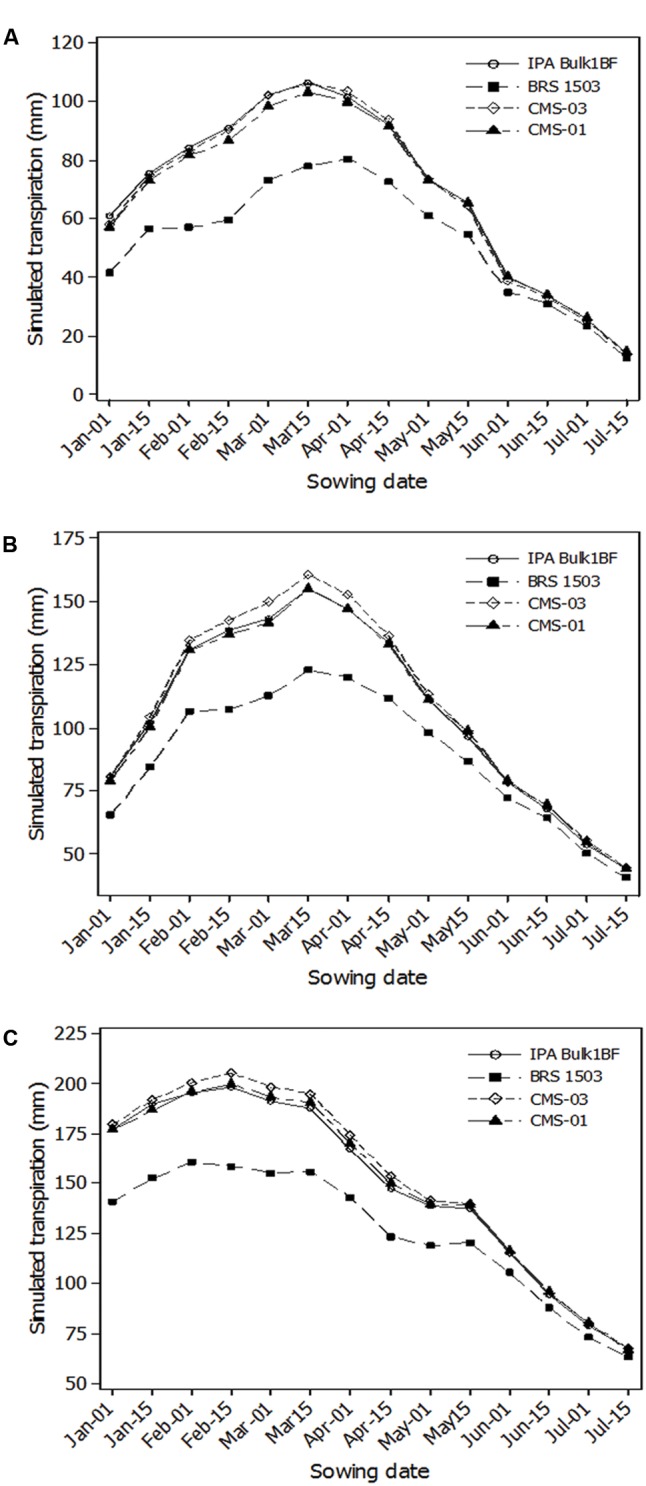
Total crop transpiration from planting to harvest for different cultivars in different sowing dates for Bom Conselho **(A)**, Nossa Senhora da Gloria **(B)**, and Sao Bento do Una **(C)**.

The total simulated transpiration for the entire season had the lowest values for the latest sowing dates and reached a maximum between 1 February and 1 April for all three locations (**Figure 3**). Usually, under water-limited conditions yield is highly correlated with transpiration.

**FIGURE 4:**
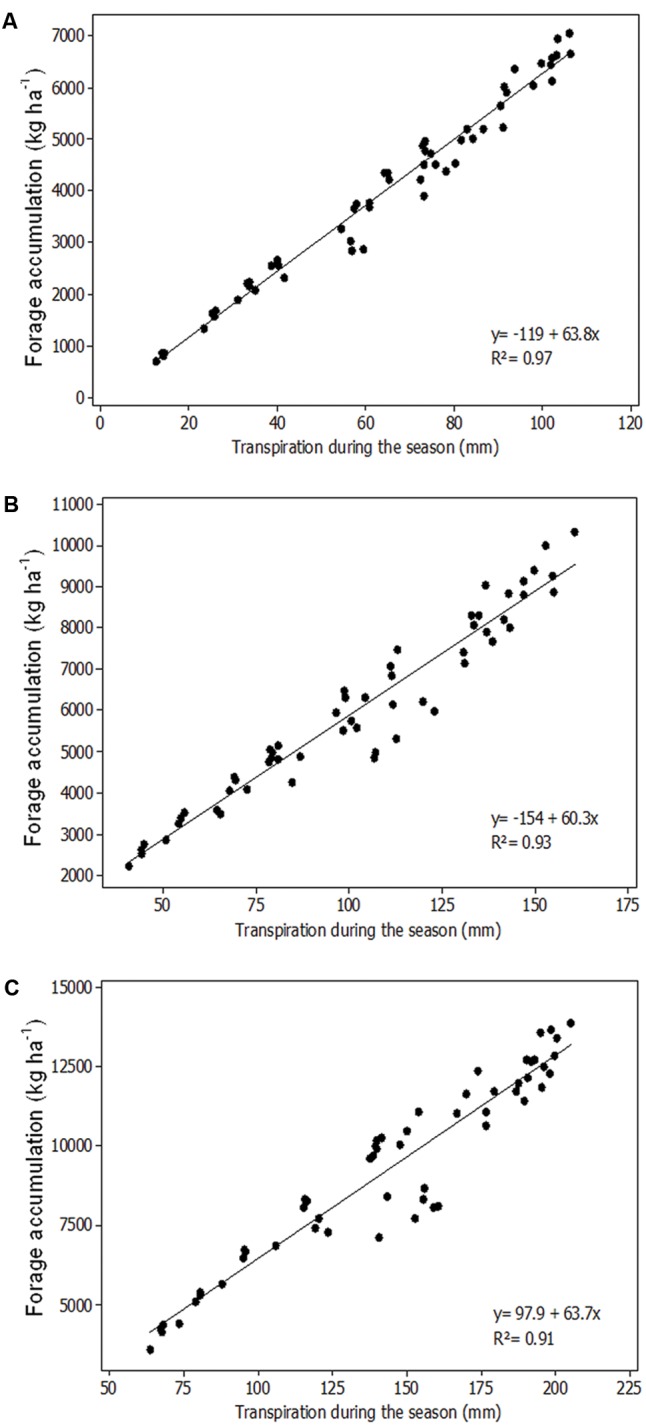
Relation between simulated total transpiration and pearl millet forage accumulation for Bom Conselho **(A)**, Nossa Senhora da Gloria **(B)**, and Sao Bento do Una **(C)**.

The coefficient of determination between simulated biomass yield and simulated total transpiration for Bom Conselho, Nossa Senhora da Gloria and Sao Bento do Una was 0.97, 0.93, and 0.97, respectively (**Figure 4**).

### Determining Sowing Window

The length of the optimum sowing window for Bom Conselho was 45 days and was shorter than the other locations. It began on 1 March and ended 15 April. For Nossa Senhora da Gloria, the cultivars influenced the period of the optimum sowing window, but the duration was 60 days for all cultivars. For the cultivars IPA Bulk1BF, CMS-03, and CMS-01, the optimum sowing window commenced on 15 February and ended on 15 April while for BRS 1501 it started on 1 March and ended 1 May. Sao Bento do Una showed the longest optimum sowing window, with a period of 75 days. The sowing window started on 15 January and finished on 1 April for all cultivars.

## Discussion

The three semi-arid locations of the present study were characterized by similarity in weather variables, except for rainfall, but the soil properties varied. For the 2011 year, as well as long term, the rainfall in Sao Bento do Una was higher and the period of the rainy season was more pronounced than in Bom Conselho and Nossa Senhora da Gloria.

The evaluated Brazilian pearl millet cultivars exhibited a shorter time from planting to anthesis than for three pearl millet varieties grown in Niger (Soler et al., 2008). Among the locations in the current study, Bom Conselho had lower simulated and measured period from planting to anthesis, likely because higher temperatures accelerated crop development and shortened the crop growth cycle. All of the indices imply that there was a good agreement between simulated and measured duration from sowing to anthesis. Based on these results, it can be concluded that the model was very robust in predicting the critical phenological growth stages.

The mean forage accumulation of 11.6 Mg ha^-1^ observed for four cultivars at three locations was greater than the range of two Brazilian cultivars (7 Mg ha^-1^) of pearl millet grown in a Brazilian sub-tropical climate (Costa et al., 2005). This may be due to greater daily heat unit accumulation in growing degree days (GDD – °C) observed during the growing season in northeast Brazil in the current study (1648 GDD – °C) compared with the Costa et al. (2005) observations in southwest Brazil (1204 GDD – °C), and better than usual rainfall in 2011, and possibly better soil fertility than the other environments. The ability of the CERES-Pearl Millet model to predict biomass in the tropical, sub-tropical and temperate environment was verified by previous studies (Shivsharan et al., 2003b; Soler et al., 2008; Dalvi et al., 2010; Hussaini and Halilu, 2013). For Sao Bento do Una, higher precipitation resulted in an increase in observed forage accumulation, which was 28 and 3% higher when compared to Bom Conselho and Nossa Senhora da Gloria, respectively. The simulated forage accumulation had the same trend as the observed since Sao Bento do Una was 36 and 8% higher than Bom Conselho and Nossa Senhora da Gloria, respectively. These significant differences in forage accumulation among locations can be attributed to several interrelated factors. One important factor to consider affecting the yield is the difference in soil properties, and particularly the difference in texture and acidity levels between the studied environments. Furthermore, there are also other interrelated factors affecting the yield under real field conditions, such as crust-prone sandy soils with low fertility, combined with unreliable and erratic rainfall, that affect the spatial variability of crops grown in semi-arid regions (Rockstrom et al., 1999).

Several studies on sowing date analysis have shown that models can be useful for this type of evaluation, compared with resource-intensive experiments (Jibrin et al., 2012; Dharmarathna et al., 2014; Andarzian et al., 2015). This study showed that long-term simulated forage accumulation of all cultivars was influenced by sowing date. For Bom Conselho a delay in sowing date from 1 January to 15 March, the average yield was increased by approximately 4.3% per week. Whereas, a delay in sowing date from 15 March to 15 July resulted in a forage accumulation reduction of about 5.1% per week.

Similar to the response at Bom Conselho, for Nossa Senhora da Gloria, delaying sowing date from 1 January to 15 March resulted in an increase of 4.9% per week, but delaying the sowing date from 15 March to 15 July decreased the forage accumulation in 4.1% per week. However, in Sao Bento do Una for the cultivars IPA Bulk1BF, CMS-03 and CMS-01, when sowing date was delayed from 1 January to 15 February the forage accumulation increased by 39 kg ha^-1^ d^-1^, but delaying from 15 February to 15 July decreased yield by 58 kg ha^-1^ d^-1^. For the cultivar BRS 1501 the yield increase was only 19 kg ha^-1^ d^-1^ when sowing date was delayed from 1 January to 15 March. A delay in sowing date from 15 March to 15 July resulted in forage accumulation decrease of 3.2% per week.

These results show that forage accumulation is influenced by cumulative intercepted solar radiance and rainfall, since in all locations solar radiation decreased and rainfall increased from January to May. This coincides with the findings of Costa et al. (2005) and Soler et al. (2008), who found that pearl millet biomass yield varies mainly due to photoperiod and water availability.

For Bom Conselho, the total simulated transpiration for the entire growing season reached a maximum between 15 March and 1 April for all four cultivars. The highest total transpiration was obtained for the cultivars IPA Bulk1BF and CMS-03 for the 15 March sowing date (106 mm). Total simulated transpiration for the entire growing season, for Nossa senhora da Gloria, showed reduced values for the late sowing dates and also showed the highest value for the 15 March sowing date for all four cultivars. Similar to other locations, for Sao Bento do Una, the total simulated crop transpiration was lowest for the final sowing dates. For the cultivars IPA Bulk1BF, CMS-03, and CMS-01 highest total transpiration was obtained for the 15 February sowing date. However, for the cultivar BRS 1501 highest total transpiration was observed for the 1 February sowing date (160 mm). Plant transpiration is directly related to yield for most cereals. A reduction in yield may occur when rainfall is insufficient to support the transpiration demand. In the present study, there were high values of the coefficient of determination between total simulated transpiration for the growing season and millet forage accumulation grown at all locations and simulated sowing dates. This indicates that water supply is one of the most important factors that limit crop production for this region, when the system is managed with relatively high inputs. These results agree with previous studies (Klaij and Vachaud, 1992; Grema and Hess, 1994; Beggi et al., 2015) that found crop water supply can be considered as the most limiting factor for millet production, when the mineral N demand is supplied. This statement is corroborated by a recent review, where Vadez et al. (2014) argued that high transpiration is indeed related to higher yield.

Therefore, the current study showed that the CSM–CERES-Pearl Millet model was able to simulate accurately growth, development, and forage accumulation for four forage pearl millet cultivars grown in three Brazilian semi-arid locations under rainfed conditions. The sowing date analysis using 15 years of climate records for Bom Conselho, Nossa Senhora da Gloria and Sao Bento do Una indicated that the best sowing dates occur before the normal forage sowing season, and the sowing window is longer than recommended by the Brazilian government for these regions. This is likely due to the absence of definitive climatic zoning, which could reduce the dependence of farmers on their individual perception of climatic factors.

In general, the results of the simulations confirmed previous field observations of pearl millet responses in this region and showed that crop simulation models can play an important role in identifying best management options for specific environmental conditions. As such, simulation models can provide farmers and policy-makers with information about forage production strategies to aid in addressing the food demands of livestock in semi-arid environments.

## Author Contributions

RS: planned the field trial, performed the statistical analysis of the field trials, and drafted the work; KB: designed crop modeling evaluation; LS: provided guidance for crop trial evaluation, and interpreted data for the work; AN and LP: established experimental design; CS: performed statistical analysis of the field trials; LG: provided guidance for crop trial evaluation.

## Conflict of Interest Statement

The authors declare that the research was conducted in the absence of any commercial or financial relationships that could be construed as a potential conflict of interest.

## References

[B1] Andarzian B. Hoogenboom G. Bannayan M. Shirali M. Andarzian B. (2015). Determining optimum sowing date of wheat using CSM-CERES-wheat model. *J. Saudi Soc. Agric. Sci.* 14 189–199.

[B2] Beggi F. Falalou H. Buerkert A. Vadez V. (2015). Tolerant pearl millet (*Pennisetum glaucum* (L.) R. Br.) varieties to low soil P have higher transpiration efficiency and lower flowering delay than sensitive ones. *Plant Soil* 389 89–108. 10.1093/jxb/erq013 20142425 PMC2837262

[B3] Costa A. C. T. Geraldo J. Pereira M. B. Pimentel C. (2005). Thermal unities and yield of pearl millet genotypes sown in two seasons. (In Portuguese, with English abstract.) *Pesqui. Agropecu. Bras.* 40 1171–1177. 10.1590/S0100-204X2005001200003

[B4] Dalvi T. D. Sabale R. N. Hazari A. K. Salunke S. S. (2010). Evaluation of sowing time for kharif pearl millet and validation by DSSAT-3.5. *J. Maharashtra Agric. Univ.* 35 385–387.

[B5] Dharmarathna W. R. S. S. Herath S. Weerakoon S. B. (2014). Changing the planting date as a climate change adaptation strategy for rice production in Kurunegala district. *Sri Lanka Sustain Sci.* 9 103–111. 10.1007/s11625-012-0192-2

[B6] Duraes F. O. M. Magalhaes P. C. Santos F. G. (2003). Fisiologia da planta do milheto. *Sete Lagoas* 1 1–16.

[B7] Fischer G. Nachtergaele F. Prieler S. van Velthuizen H. T. Verelst L. Wiberg D. (eds). (2008). “HWSD global soil quality – Constraints on nutrient availability,” in *Global Agro-Ecological Zones Assessment for Agriculture: GAEZ 2008* (Rome: IIASA).

[B8] Grema A. K. Hess T. M. (1994). Water balance and water use of pearl millet-cowpea intercrops in north east Nigeria. *Agric. Water Manage.* 26 169–185. 10.1016/0378-3774(94)90056-6

[B9] Hill G. M. Utley P. R. Gates R. N. Hanna W. W. Johnson J. C. (1999). Pearl millet silage for growing beef heifers and steers. *J. Produ. Agric.* 12 653–658. 10.2134/jpa1999.0533

[B10] Hoogenboom G. Jones J. W. Boote K. J. (1992). Modeling growth, development, and yield of grain legumes using Soygro, Pnutgro, and Beangro: a review. *Trans. ASAE* 35 2043–2055. 10.13031/2013.28833

[B11] Hoogenboom G. Jones J. W. Wilkens P. W. Porter C. H. Boote K. J. Hunt L. A. (2015). *Decision Support System for Agrotechnology Transfer (DSSAT).* Washington, DC: DSSAT Foundation, Prosser.

[B12] Hussaini M. S. Halilu A. (2013). Evaluation of DSSAT crop model for the prediction of irrigated pearl millet yield. *Int. J. Eng. Sci.* 2 450–453.

[B13] Jamieson P. D. Porter J. R. Wilson D. R. (1991). A test of the computer simulation model ARCWHEAT1 on wheat crops grown in New Zealand. *Field Crops Res.* 27 337–350. 10.1016/0378-4290(91)90040-3

[B14] Jibrin J. M. Kamara A. Y. Ekeleme F. (2012). Simulating planting date and cultivar effects on dryland maize production using CERES-maize model. *Afr. J. Agric. Res.* 7 5530–5536.

[B15] Jones J. W. Hoogenboom G. Porter C. H. Boote K. J. Batchelor W. D. Hunt L. A. (2003). DSSAT Cropping system model. *Eur. J. Agron.* 18 235–265. 10.1016/S1161-0301(02)00107-7

[B16] Jones J. W. Keating B. A. Porter C. H. (2001). Approaches to modular model development. *Agric. Syst.* 70 421–443. 10.1016/S0308-521X(01)00054-3

[B17] Klaij M. C. Vachaud G. (1992). Seasonal water balance of a sandy soil in Niger cropped with pearl millet, based on profile moisture measurements. *Agric. Water Manage.* 21 313–330. 10.1016/0378-3774(92)90053-Y

[B18] Loague K. Green R. E. (1991). Statistical and graphical methods for evaluating solute transport models: overview and application. *J. Contam. Hydrol.* 7 51–73. 10.1016/0169-7722(91)90038-3

[B19] Maiti R. Wesche-Ebeling P. (1997). *Pearl Millet Science.* Enfield, CT: Science Publishers Inc.

[B20] Martins L. M. V. Xavier G. R. Rangel F. W. Ribeiro J. R. A. Neves M. C. P. Morgado L. B. (2003). Contribution of biological nitrogen fixation to cowpea: a strategy for improving grain yield in the semi-arid region of Brazil. *Biol. Fertil. Soils* 38 333–339. 10.1007/s00374-003-0668-4

[B21] Matthews R. Stephens W. Hess T. Middleton T. Graves A. (2002). Applications of crop/soil simulation models in tropical agricultural systems. *Adv. Agronomy* 76 31–124. 10.1016/S0065-2113(02)76003-3 22505776

[B22] Messman M. A. Weiss W. P. Henderlong P. R. Shokey W. L. (1992). Evaluation of pearl millet and field peas plus triticale silages for midlactation dairy cows. *J. Dairy Sci.* 2 2769–2776. 10.3168/jds.S0022-0302(92)78040-0 1430482

[B23] Pale S. Mason S. C. Galusha T. D. (2003). Planting time for early-season pearl millet and grain sorghum in Nebraska. *Agron. J.* 95 1047–1053. 10.2134/agronj2003.1047

[B24] Pereira Filho I. A. Pereira A. S. Coelho A. M. Casela C. R. Karam D. Rodrigues J. A. S. (2003). Manejo da cultura do milheto. *Sete Lagoas* 1 1–17.

[B25] Priestley C. H. B. Taylor R. J. (1972). On the assessment of surface heat flux and evaporation using large scale parameters. *Mon. Weather Rev.* 100 81–92. 10.1175/1520-0493(1972)100<0081:OTAOSH>2.3.CO;2

[B26] Rockstrom J. Barron J. Brouwer J. Galle S. De Rouw A. (1999). On-farm spatial and temporal variability of soil and water in pearl millet cultivation. *Soil Sci. Soc. Am. J.* 63 1308–1319. 10.2136/sssaj1999.6351308x

[B27] Rodrigues J. A. S. Pereira Filho I. A. (2010). Cultivo do milheto. *Sete* *Lagoas* 1 1–3.

[B28] Ruiz-Nogueira B. Boote K. J. Sau F. (2001). Calibration and use of CROPGRO-soybean model for improving soybean management under rainfed conditions. *Agric. Syst.* 68 151–173. 10.1016/S0308-521X(01)00008-7

[B29] Santos H. G. Jacomine P. K. T. Anjos L. H. C. Oliveira V. A. Lumbreras J. F. Coelho M. R. (2013). *Sistema Brasileiro de Classificação de Solos.* Brasília: Embrapa.

[B30] Saseendran S. A. Ma L. Nielsen D. C. Vigil M. F. Ahuja L. R. (2005). Simulating planting date effects on corn production using RZWQM and CERES-Maize Models. *Agron. J.* 97 58–71. 10.2134/agronj2005.0058

[B31] Saxton K. E. Rawls W. J. Romberger J. S. Papendick R. I. (1986). Estimating generalized soil-water characteristics from texture. *Soil Sci. Soc. Am. J.* 50 1031–1036. 10.2136/sssaj1986.03615995005000040054x

[B32] Shivsharan P. N. Sabale R. N. Varshneya M. C. (2003a). Validation of DSSAT 3.5 for summer season pearl millet. *J. Maharashtra Agric. Univ.* 28 173–176.

[B33] Shivsharan P. N. Sable R. N. Varshneya M. C. More D. B. (2003b). Validation studies of DSSAT-3.5 for pearl millet in summer season in the Pune region of Maharashtra state. *J. Agrometeorol.* 5 68–72.

[B34] Singh B. R. Singh D. P. (1995). Agronomic and physiological responses of sorghum, maize and pearl millet to irrigation. *Field Crops Res.* 42 57–67. 10.1016/0378-4290(95)00025-L

[B35] Soler C. M. T. Maman N. Zhang X. Mason S. C. Hoogenboom G. (2008). Determining optimum planting dates for pearl millet for two contrasting environments using a modelling approach. *J. Agric. Sci.* 146 445–459. 10.1017/S0021859607007617

[B36] Soler C. M. T. Sentelhas P. C. Hoogenboom G. (2007). Application of the CSM-CERES-Maize model for planting date evaluation and yield forecasting for maize grown off-season in a subtropical environment. *Eur. J. Agron.* 27 165–177. 10.1016/j.eja.2007.03.002

[B37] Stackhouse P. W. Jr. Westberg D. Hoell J. M. Chandler W. S. Zhang T. (2015). *Prediction Of Worldwide Energy Resource (POWER) – Agroclimatology Methodology, Version 1.0.2.* Washington, DC: The National Aeronautics and Space Administration.

[B38] Tsuji G. Y. Hoogenboom G. Thornton P. K. (1998). *Understanding Options for Agricultural Production.* Honolulu, HI: Springer.

[B39] Vadez V. Kholova J. Medina S. Kakkera A. Anderberg H. (2014). Transpiration efficiency: new insights into an old story. *J. Exp. Bot.* 65 6141–6153. 10.1093/jxb/eru040 24600020

[B40] Van Ittersum M. K. Leffelaar P. A. Van Keulen H. Kropff M. J. Bastiaans L. Goudriaan J. (2003). On approaches and applications of the Wageningen crop models. *Eur. J. Agron.* 18 201–234. 10.1016/S1161-0301(02)00106-5

[B41] Willmott C. J. Ackleson S. G. Davis R. E. Feddema J. J. Klink K. M. Legates D. R. (1985). Statistics for the evaluation and comparison of models. *J. Geophys. Res. Oceans* 90 8995–9005. 10.1029/JC090iC05p08995

[B42] Zadoks J. C. Chang T. T. Konzak C. F. (1974). A decimal code for the growth stages of cereals. *Weed Res.* 14 415–421. 10.1111/j.1365-3180.1974.tb01084.x

